# Methicillin and inducible clindamycin resistance in clinical *Staphylococcus aureus* isolates: a cross-sectional study from Northwest Ethiopia

**DOI:** 10.3389/fmicb.2025.1569242

**Published:** 2025-06-13

**Authors:** Zemen Addis, Yibeltal Aschale, Abebe Fenta, Zigale Hibstu Teffera, Abateneh Melkamu, Abeba Tigab, Tebelay Dilnessa

**Affiliations:** ^1^Department of Medical Laboratory Sciences, College of Medicine and Health Sciences, Debre Markos University, Debre Markos, Ethiopia; ^2^Department of Medical Laboratory Sciences, College of Health Sciences, Debark University, Debark, Ethiopia; ^3^Medical Microbiology Laboratory Team, Debre Markos Comprehensive Specialized Hospital, Debre Markos, Ethiopia

**Keywords:** antimicrobial resistance, prevalence, *S. aureus*, methicillin resistance, inducible clindamycin resistance, Ethiopia

## Abstract

**Background:**

*Staphylococcus aureus* is a major pathogenic bacterium associated with high morbidity and mortality worldwide. It exhibits resistance to multiple antibiotics, complicating treatment options. Despite its clinical significance, there is limited data on the prevalence of *S. aureus* infections and the patterns of methicillin and inducible clindamycin resistance, particularly in Ethiopia. Understanding these resistance trends is essential for guiding appropriate therapy and improving patient outcomes.

**Objective:**

To assess the prevalence of *S. aureus*, methicillin and inducible clindamycin resistance patterns, and associated factors among patients with suspected bacterial infection at Debre Markos Comprehensive Specialized Hospital, Northwest Ethiopia.

**Method:**

A hospital-based cross-sectional study was conducted among patients suspected of bacterial infections from 10 June 2023 to 28 February 2024. Blood, wound swab, cerebrospinal fluid, urine, eye swab, synovial fluid, ear swab, and pleural fluid were collected aseptically and inoculated onto appropriate media. *S. aureus* was identified based on colony morphology, Gram staining, DNase test and biochemical tests. Antimicrobial susceptibility testing was performed on the isolates using the disk diffusion and the D-test techniques based on CLSI guideline. Data were entered into SPSS version 26 for analysis. Logistic regression was applied to assess the relationship between predictors and the outcome variable. A *P*-value of ≤ 0.05 with a 95% CI was considered statistically significant.

**Results:**

Among the 339 study participants, 38 (11.2%) (95% CI: 8–15) tested positive for *S. aureus*. Of these isolates, 14/38 (36.9%) were resistant to methicillin, 5/38 (13.1%) isolates were resistance to clindamycin by routine disk diffusion test whereas 10/38 (26.3%) isolates exhibited inducible clindamycin resistance using the D-test. The level of multidrug resistance was noted in 10/38 (26.3%) of the isolates. Significant factors associated with *S. aureus* infection included illiteracy (AOR = 13.51; 95% CI: 3.56–21.90; *P* = 0.018), having larger family size (AOR = 12.14; 95% CI: 2.38–20.43; *P* = 0.024), and income level of less than 3,000 ETB (AOR = 6.20; 95% CI: 1.03–30.09; *P* = 0.046).

**Conclusion:**

The study revealed an 11.2% occurrence of *S. aureus* among the study participants, with a substantial proportion exhibiting methicillin resistance (36.9%) and inducible clindamycin resistance (26.3%). These findings highlight a higher burden of clindamycin resistance in MRSA, underscoring the need for routine D-test screening to guide appropriate antibiotic therapy. Additionally, factors such illiteracy, low income and residing in rural areas were associated with *S. aureus* infection. Targeted health education initiatives should be implemented, especially in rural areas and among populations with low literacy levels, to improve hygiene practices and reduce transmission.

## 1 Introduction

*Staphylococcus aureus* was first identified in 1880 by the Scottish surgeon Sir Alexander Ogston, who isolated it from the pus of surgical abscesses. This organism is classified as a Gram-positive bacterium and exhibits an aerobic to facultative anaerobic lifestyle, commonly colonizing the skin, nares, and axillae of humans (Deurenberg and Stobberingh, 2008). *S. aureus* is a major human pathogen responsible for a wide spectrum of infections ranging from superficial skin conditions to life-threatening systemic diseases such as bacteremia, pneumonia, and endocarditis (Tong et al., 2015). Globally, the rise of antibiotic-resistant strains, particularly Methicillin-Resistant *S. aureus* (MRSA), has become a critical public health challenge due to its limited treatment options and association with increased morbidity, mortality, and healthcare costs (Lee et al., 2018).

*S. aureus* is a leading cause of both hospital and community-acquired infections, which include a variety of serious conditions such as: urinary tract, bone and joint infections, meningitis, bacteremia, endocarditis, skin and soft tissue, surgical site, and toxic shock syndrome (Sit et al., 2017; You et al., 2017). Infections caused by *S. aureus* are associated with significant morbidity, mortality, and economic burden. For instance, in 2007, more than 170,000 individuals in Europe experienced bloodstream infections due to MRSA, resulting in approximately 5,400 fatalities and an estimated economic cost of €380 million. In the United States, the Centers for Disease Control and Prevention (CDC) reported 11,285 deaths and over 80,000 bloodstream MRSA infections in 2011 (Alli et al., 2011).

Methicillin-resistant *S. aureus* has become a major public health concern due to its resistance to multiple antibiotics, complicating treatment and leading to worse clinical outcomes. According to the World Health Organization, MRSA is categorized as a “high priority” pathogen for research and development of new antibiotics (WHO, 2017). Globally, MRSA accounts for about 20–50% of *S. aureus* infections depending on the region, with the highest prevalence reported in parts of Asia, Africa, and Latin America (Tong et al., 2015). Studies have found MRSA prevalence rates ranging from 25% to 50% among *S. aureus* isolates from various hospitals in Ethiopia (Kejela and Bacha, 2013; Tadesse et al., 2017).

Methicillin-resistant *S. aureus* is commonly associated with healthcare settings, including recent hospitalization, surgical procedures, the presence of invasive devices, and residence in long-term care facilities (David and Daum, 2010). Prior or prolonged use of antibiotics, particularly β-lactams and fluoroquinolones, as well as poor hygiene practices, have been shown to increase the risk of MRSA colonization (Tacconelli et al., 2008). Additionally, crowded living conditions, low socioeconomic status, and environments involving close physical contact-such as prisons or military settings-have been linked to higher MRSA prevalence (Miller and Diep, 2008). Similarly, hospitalization and frequent use of antibiotics, particularly macrolides such as erythromycin, can promote the selection of bacterial strains carrying *erm* genes, which are responsible for inducible macrolide-lincosamide-streptogramin B (iMLS) resistance (Deotale et al., 2010; Leclercq, 2002). These *erm* genes- specifically *erm(A)*, *erm(B)*, and *erm(C)*-mediate iMLS resistance by methylating the 23S rRNA target site of the bacterial ribosome (Fiebelkorn et al., 2003).

For the treatment of methicillin-resistant *S. aureus* isolates, antibiotics such as vancomycin, linezolid, quinupristin, and dalfopristin have been the preferred options. However, the increasing reports of resistance to these antibiotics have raised concerns regarding their effectiveness (Cong et al., 2020). In response to this challenge, physicians have turned to the macrolide-lincosamide-streptogramin B (MLSB) family of antibiotics as an alternative treatment for MRSA infections. Among these, clindamycin remains an important antibiotic for treating *S. aureus* infections, specifically in penicillin-allergic patients. However, the emergence of inducible clindamycin resistance, mediated by the erm genes, threatens its clinical utility (Gemmell et al., 2006). This resistance may not be detected by standard susceptibility testing unless a specific D-test is performed, leading to potential treatment failures (Khashei et al., 2018; Leclercq, 2002).

The detection of inducible clindamycin resistance is essential and is commonly performed using the D-test, which identifies isolates with the potential to develop resistance during therapy. While several studies have reported the prevalence of MRSA globally, data on the local prevalence of both methicillin and inducible clindamycin resistance among *S. aureus* isolates remain scarce in Ethiopia (Kejela and Bacha, 2013; Tadesse et al., 2017). Variations in resistance patterns across different geographic regions highlight the need for continuous local surveillance to inform empirical treatment and infection control measures. Inducible clindamycin resistance (iMLSB phenotype) in *S. aureus* is a significant concern in Ethiopia, as it can lead to treatment failures if not properly identified.

However, there is limited information on the prevalence of *S. aureus*, methicillin and inducible clindamycin resistance, along with associated factors in Ethiopia. In Ethiopia, antibiotic resistance surveillance faces significant challenges, including limited laboratory capacity, shortages of trained personnel, fragmented surveillance systems, and resource constraints (Tadesse et al., 2017; WHO, 2014). Moreover, the prioritization of other communicable diseases, lack of political commitment, and widespread misuse of antibiotics further hinder the establishment of comprehensive antimicrobial resistance (AMR) data collection systems (WHO, 2015). Therefore, this study aimed to assess the prevalence of *S. aureus*, methicillin resistance, and inducible clindamycin resistance, along with the associated factors among patients at Debre Markos Comprehensive Specialized Hospital, Northwest Ethiopia.

## 2 Materials and methods

### 2.1 Study area and setting

The study was conducted at Debre Markos Comprehensive Specialized Hospital (DMCSH), which is found in Debre Markos town, the capital of East Gojjam zone, located 302 km Northwest of Addis Ababa, the capital city of Ethiopia, and 264 km Southeast of Bahir Dar, the capital of Amhara National Regional State. It is one of the oldest public hospitals in the country which was established in 1957. It provides health services for approximately 255,248 patients per year from a catchment population of about 5 million people. It gives service to East Gojjam, West Gojjam, Awi zone, and some parts of the Oromia region (Asmare et al., 2021).

### 2.2 Study design and period

A hospital-based cross-sectional study was conducted from 10 June 2023 to 28 February 2024.

### 2.3 Study population

All patients suspected of having bacterial infections at DMCSH during the study period.

### 2.4 Eligibility criteria

#### 2.4.1 Inclusion criteria

1. Patients of all age groups and genders suspected of bacterial infections attending Debre Markos Comprehensive Specialized Hospital during the study period.
2. Patients who provided appropriate clinical specimens [e.g., blood, wound swabs, ear swab, urine, cerebrospinal fluid (CSF)] for bacterial culture and sensitivity testing.
3. Patients who gave informed consent (or assent, with parental/guardian consent for minors) to participate in the study.

#### 2.4.2 Exclusion criteria

1. Patients who had received antibiotic therapy within two weeks prior to sample collection, to avoid altered culture results.
2. Patients with incomplete clinical information or whose samples were improperly collected.
3. Repeat isolates from the same patient (only the first isolate was included to avoid duplication). Duplicate isolates were avoided through match on patient identification. Additionally, laboratory information system (LIS) data platforms allowed tracking of isolates and patient data.

### 2.5 Sample size determination and sampling technique

The sample size was calculated by using single population proportion formula based on the assumption of a 95% confidence interval (Zα/2 = 1.96), 4% margin of error (to increase sample size), and prevalence of 17% from a previous study in Gondar town, Ethiopia (Ambachew et al., 2022).

n=(Zα/2)=2P⁢(1-P)d2(1.96)2⁢0.17*0.83(0.04)2=339

Where: n = sample size; p = prevalence of *S. aureus* taken from Gondar, Ethiopia (17%) = 0.17, q = (1–p) = 0.83, z = 1.96, critical value; d = 0.04, precision (margin of error). Thus, setting these values into the formula, the sample size was 339. Therefore, a consecutive convenience sampling technique was used to enroll the study participants. We included all accessible participants that met the inclusion criteria and presented during the study period.

### 2.6 Operational definitions

Methicillin-resistant *S. aureus* (MRSA): *S. aureus* with a zone of inhibition ≤ 21 mm for cefoxitin on MHA after 16–18 h of incubation (CLSI, 2022).

Inducible clindamycin resistance (iMLS_*B*_) phenotype: Resistant to erythromycin (zone size ≤ 13 mm) and susceptible to clindamycin (zone size ≥ 21 mm) with a D-shaped zone of inhibition around the clindamycin disk (Seifi et al., 2012).

Constitutive clindamycin resistance (cMLSB) phenotype: *S. aureus* isolates were resistant to both drugs erythromycin (ERY) and clindamycin (CLN) (Leclercq, 2002).

Macrolide-streptogramin B (MS) phenotype: *S. aureus* isolates exhibited resistance to ERY and were sensitive to CLN with no D-shaped zone of inhibition (Ungureanu, 2010).

Sensitive (S) phenotype: *S. aureus* isolates were sensitive to both erythromycin (ERY) and clindamycin (CLN) (CLSI, 2022).

Clinical samples: Clinical samples in this study refer to blood, urine, wound swab, CSF, synovial fluid, ear swab, eye swabs and pleural fluid.

Multidrug resistance (MDR): *S. aureus* resistance to three or more antimicrobials belonging to different classes of drugs (Magiorakos et al., 2012).

### 2.7 Data collection and processing

#### 2.7.1 Socio-demographic and clinical related data collection

Data collectors received one day of on-site training on the procedures for collecting relevant data. A structured questionnaire was used to gather information on socio-demographic characteristics, clinical details, and potential factors associated with the condition under study. The questionnaire was initially prepared in English, then translated into Amharic, and subsequently back-translated into English to ensure consistency and accuracy. The validity of the questionnaire was assessed through content validity, face validity, construct validity, pilot testing, and reliability testing.

#### 2.7.2 Specimen collection and transportation

Clinical specimens including blood, wound swab, cerebrospinal (CSF), urine, eye swab, synovial fluid, ear swabs and pleural fluid were collected aseptically and transported to the medical microbiology laboratory section of the hospital. A strict aseptic technique was followed to collect the sample for culture and dispensed with great care to avoid contaminating the specimen and culture medium. If delay was unavoidable to process; blood, synovial fluid, pleural fluid samples were stored at room temperature and CSF samples were stored in the incubator at body temperature while ear swab, eye swab, urine and wound swabs were stored in refrigerator.

Blood samples were collected before initiation of antimicrobial therapy, directly into blood culture bottles (5–10 mL per bottle), and transported without delay. Cerebrospinal samples were obtained by lumbar puncture using sterile technique, following skin antiseptics with 1–2% tincture of iodine and 70% alcohol. Urine samples were collected via clean-catch midstream, catheterization, or as a first-morning specimen in sterile containers. Wound swabs were collected after decontaminating the surrounding skin and expressing pus from the lesion. Pleural fluid was aspirated aseptically via thoracentesis. Ear swabs were taken from the external canal after cleaning the outer ear. Eye swabs were collected from the conjunctival sac using sterile moistened swabs. Synovial fluid was aspirated by arthrocentesis under sterile conditions following local antiseptics.

### 2.8 Isolation and identification of *S. aureus*

Upon arrival to medical microbiology laboratory section of the hospital, all clinical specimens except blood samples, were inoculated onto mannitol salt agar (MSA) and blood agar plates (BAP) using sterilized wire loops to obtain discrete colonies. Blood samples were first inoculated into tryptone soya broth (TSB) and incubated aerobically at 37°C for up to one week, with daily inspection for signs of bacterial growth every 24 h. Positive blood cultures were subsequently sub-cultured onto MSA and BAP. The MSA plates were incubated aerobically at 37°C for 24 h, while the BAPs were incubated at 37°C for 24 h in a candle jar to create a microaerophilic environment. After incubation, plates were examined for the presence of golden-yellow colonies on MSA and large β-hemolytic colonies on BAP, indicative of potential *S. aureus* isolates. The suspected colonies were further confirmed through Gram staining, DNase agar test, catalase test, and coagulase test (Versalovic, 2011).

### 2.9 Antimicrobial susceptibility testing

Antimicrobial susceptibility testing of *S. aureus* was performed by using the Kirby–Bauer disk diffusion technique on the Mueller–Hinton Agar (MHA) according to the Clinical and Laboratory Standards Institute (CLSI). The antimicrobial agents used were cefoxitin (FOX, 30 μg), clindamycin (CLN, 2 μg) and erythromycin (ERY, 15 μg). Cefoxitin disk was used to characterize MRSA isolates. Other antibiotics include: ciprofloxacin (CIP, 5 μg), gentamicin (GEN, 10 μg), azithromycin (AZM, 15 μg), clindamycin (CLN, 2 μg), trimethoprim/sulfamethoxazole (SXT, 1.25/23.75 μg), penicillin (P, 10 units), tetracycline (T, 30 μg), and nitrofurantoin (NIF, 30 μg).

A sterile wire loop was used to transfer a loopful of bacteria (2–3 identical colonies) from a pure culture colony to a test tube containing 5 ml of normal saline. The mixture was gently agitated to create a uniform suspension. The turbidity of the bacterial suspension was standardized using the 0.5 McFarland standard. Sterile cotton swabs were then immersed in the suspension, and excess fluid was removed by gently rotating the swab against the inner surface of the tube. The bacterial suspension was inoculated over the entire surface of Mueller–Hinton agar (MHA). Antimicrobial disks were placed on the agar, which was then incubated at 37 °C for 18–24 h. A zone of inhibition was measured in millimeters using a ruler around each antimicrobial disk. The results was reported as sensitive, resistant, or intermediate according to the CLSI, 2022 guideline (CLSI, 2022).

#### 2.9.1 Detection of methicillin resistance *S. aureus* (MRSA)

The MRSA isolates were confirmed phenotypically using cefoxitin disk (30 μg). The plates were incubated aerobically at 35°C for 18 h on Mueller Hinton agar. *S. aureus* which was yielded a zone diameter of 21 mm or less with cefoxitin disk was phenotypically confirmed as MRSA. *S. aureus* ATCC 25923 was used as quality control (Morris and Simner, 2019).

#### 2.9.2 D-test and interpretations

All isolates were subjected to D-test on Mueller–Hinton agar plate for phenotypic detection of clindamycin resistance by using erythromycin (15 μg) and clindamycin (2 μg) disks that were positioned 15–20 mm apart from one another. Positive inducible clindamycin resistance (iMLSB phenotype) was defined as the appearance of a flattened clindamycin zone between clindamycin and erythromycin producing a D-shape with erythromycin resistance (D-test positive). *S. aureus* isolates showing circular zones of inhibition with a diameter of ≤ 13 mm for erythromycin (ERY) and ≥ 21 mm for clindamycin (CLN) without a D-shaped zone along ERY was interpreted as negative for inducible resistance (D-test negative) (Shittu and Lin, 2006).

### 2.10 Data quality assurance

Before the use of any reagents, antibiotic disks, and culture media, appropriate storage conditions and the expiration date were checked. The pre-test was carried out at Finote Selam Hospital to check for completeness and consistency of the questionnaire. The culture media, Gram staining reagents, and antibiotic disks were checked for expiry dates prior to use. Standard operating procedures were followed for all the methods described above. The prepared culture media, biochemical test, and antimicrobial susceptibility tests were checked by inoculating the reference strain *S. aureus* (ATCC-25923). The sterility and performance of the prepared culture media was also checked.

### 2.11 Data analysis and interpretations

Data was checked for its completeness before entering for analysis. It was cleaned, coded, and entered EpiData 3.1 software, which was then be exported, to Statistical Package for Social Sciences software (SPSS) version 26 for analysis. Descriptive analysis was conducted to organize, summarize, and interpret the data using measures of prevalence and percentage distributions. Bivariable logistic regression analysis was employed to examine the association between independent variables including educational attainment, family size, household income, and others with *S. aureus* infections. A *P*-value ≤ 0.25 was used as a cut-off for inclusion into the multivariate model. Variables with *P*-value less than or equal to 0.25 in the bivariate analysis were jointly entered into a multivariate logistic regression analysis. A *P*-value ≤ 0.05 with 95% CI was considered statistically significant. We assessed multicollinearity among predictor variables using the Variance Inflation Factor (VIF) prior to conducting multivariate logistic regression. Key variable pairs, such as educational status and income, age group and chronic disease, and others were examined. The highest VIF observed was 4 (for educational status and income), which is below the accepted threshold, indicating no significant multicollinearity concerns.

### 2.12 Ethics approval and consent to participate

Ethical approval was obtained from Institutional Health Research Ethics and Review Committee (IRERC) of College of Medicine and Health Sciences, Debre Markos University (Ref. No: HSR/RCS/141/11/12), dated on 04/04/2023. Written permission was obtained from Debre Markos Comprehensive Specialized Hospital for the data collection process. Written informed assent and consent was obtained from the parents/guardians and study participants, respectively. Participation was only based on voluntarism. Confidentiality and anonymity were also maintained.

## 3 Results

### 3.1 Socio-demographic and clinical characteristics of the study participants

**FIGURE 1:**
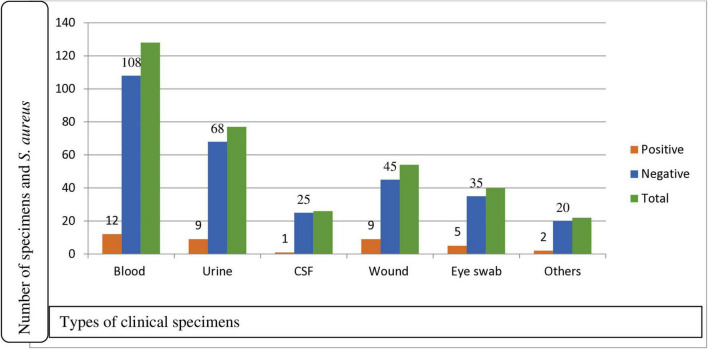
Prevalence of *S. aureus* for clinical specimens among participants suspected for bacterial infection at Debre Markos Comprehensive Specialized Hospital, Northwest Ethiopia, 2024 (*N* = 339). CSF, cerebrospinal fluid; Others: ear swab, synovial fluid, pleural fluid.

A total of 339 patients suspected of having bacterial infections were enrolled in this study, of which 179/339 (52.8%) were males. Eight types of clinical samples were included in the culture analysis. The majority of participants were in the age group of 31–50 years, comprising 114/339 (33.6%) individuals. In terms of residence, 181/339 (53.4%) participants were urban dwellers. Regarding educational status, 43/339 (12.7%) participants reported being unable to read and write (Table 1). The most frequently collected specimens were blood (128), urine (77), and wound swabs (54). Among these, *S. aureus* was isolated in 38/339 (11.2%) cases, while 301/339 (88.8%) samples showed no growth of *S. aureus* (Figure 1).

**TABLE 1:** Prevalence of *S. aureus* among study participants along with sociodemographic factors suspected for bacterial infection at Debre Markos Comprehensive Specialized Hospital, Northwest Ethiopia, 2024 (*N* = 339).

Socio-demographic variables		Culture status of *S. Aureus*			Total study participants *n* (%)
		Positive		Negative, *n* (%)	
		*n* (%)	95% CI		
Sex	Male	22 (57.9)	40.0–75.1	157 (52.2)	179 (52.8)
	Female	16 (42.1)	36.2–55.7	144 (47.8)	160 (47.2)
Age group	< 1 year	3 (7.9)	0.8–14.0	73 (24.3)	76 (22.4)
	1–10 years	3 (7.9)	2.4–19.9	23 (7.6)	26 (7.7)
	11–20 years	1 (2.6)	0.1–21.9	23 (7.6)	24 (7.1)
	21–30 years	6 (15.8)	7.4–23.8	45 (15.0)	51 (15.0)
	31–50 years	14 (36.8)	22.9–40.1	100 (33.2)	114 (33.6)
	≥ 51 years	11 (29)	22.0–35.0	37 (12.3)	48 (14.2)
Residence	Urban	14 (36.8)	26.9–41.6	167 (55.5)	181 (53.4)
	Rural	24 (63.2)	59.6–70.8	134 (44.5)	158 (46.6)
Educational status	Illiterate	12 (31.6)	24.5–41.3	31 (10.3)	43 (12.7)
	Primary school	12 (31.6)	20.7–41.3	63 (20.1)	75 (22.1)
	Secondary school	8 (21.0)	14.8–31.9	52 (17.3)	60 (17.7)
	College and above	6 (15.8)	2.8–26.7	155 (51.5)	161 (47.5)
Occupation	Government employeed	4 (10.5)	0.2–17.4	103 (34.2)	107 (31.6)
	Farmer	20 (52.6)	46.2–65.8	88 (29.2)	108 (31.9)
	Self-employeed	3 (7.9)	0.4–19.6	27 (9.0)	30 (8.8)
	Daily laborer	2 (5.3)	0.2–20.7	13 (4.3)	15 (4.4)
	Housewife	3 (7.9)	1.2–22.8	22 (7.3)	25 (7.4)
	Merchant	4 (10.5)	1.6–19.8	45 (14.9)	49 (14.5)
	Others	2 (5.3)	0.5–12.9	3 (1.0)	5 (1.5)
Family size	1–3	6 (15.8)	11.0–28.1	125 (41.5)	131 (38.6)
	4–6	29 (76.3)	63.8–80.7	168 (55.8)	197 (58.1)
	> 6	3 (7.9)	1.0–13.5	8 (2.7)	11 (3.2)
Income (ETB)	≤ 3,000	20 (52.6)	45.5–64.5	60 (19.9)	80 (23.6)
	3,001–6,000	14 (36.8)	24.6–43.6	140 (39.8)	154 (45.4)
	≥ 6,001	4 (10.6)	8.1–27.5	101 (29.8)	105 (31.0)
Types of specimens	Blood	12 (31.6)	24.4–40.3	108 (35.9)	128 (37.7)
	Urine	9 (23.7)	15.6–32.8	68 (22.6)	77 (22.7)
	Wound	9 (23.7)	15.9–28.4	45 (14.9)	54 (15.9)
	Eye swab	5 (13.2)	8.5–22.5	35 (11.6)	40 (11.8)
	CSF	1 (2.6)	0.1–11.3	25 (8.3)	26 (7.6)
	Others	2 (5.2)	0.2–21.1	20 (6.7)	22 (6.5)
Total		38 (11.2)	8.0–15.0	301 (88.8)	339 (100)

1$ = 131.6 ETB; CSF, cerebrospinal fluid; Others: ear swab, synovial fluid, pleural fluid.

### 3.2 Prevalence of *S. aureus* infections

Of a total of 339 participants, 38 (11.2%) (95% CI: 8–15) were found to have *S. aureus* isolates. Among the isolates, males accounted for the highest proportion, 22/38 (57.9%; 95% CI: 40.0–75.1). In terms of residence, rural dwellers accounted for 24/38 (63.6%) isolates, while participants who were illiterate had an isolation rate of 12/38 (31.6%). The majority of the isolates were recovered from patients aged 31–50 years, accounting for 14/38 (36.8%) isolates, followed by those aged greater or equal to 51 years, 11/38 (29%) isolates. Blood samples yielded the highest number of *S. aureus* isolates, 12/128 (9.4%). Wound swab and urine yielded, 9/54 (16.7%) and 9/77 (11.7%) isolates each, indicating infection or colonization, respectively. Eye swabs 5/40 (12.5%), CSF 1/26 (3.8%), and other specimens 2/22 (10%) contributed fewer isolates, but still reflect the pathogen’s presence in diverse clinical conditions. Of the total isolates, 12/38 (31.6%) were obtained from blood samples, followed by 9/38 (23.7%) each from wound and urine samples (Table 1).

### 3.3 Antimicrobial susceptibility pattern of *S. aureus*

The antimicrobial susceptibility testing of 38 *S. aureus*, azithromycin exhibited the highest sensitivity at 32/38 (84.2%), followed by clindamycin 28/38 (73.8%) and ciprofloxacin 27/38 (71.0%). Conversely, penicillin and gentamicin showed the highest resistance rates, at 19/38 (50.0%) and 16/38 (42.1%), respectively (Table 2). Of the total *S. aureus* isolates, 14/38 (36.8%) were methicillin-resistant *S. aureus* (MRSA), and 24/38 (63.2%) were methicillin-sensitive *S. aureus* (MSSA). Among the MRSA cases, 9/14 (64.3%) were males and 5/14 (35.7%) were females. For MSSA, 14/24 (58.3%) were male and 10/24 (41.7%) were females. Overall, males accounted for 22/38 (57.9%) of the total isolates, and females for 16/38 (42.1%). MRSA was most commonly isolated from individuals aged 31–50 years 6/14 (42.8%), followed by those older than 51 years 4/14 (28.6%). No MRSA was identified in the 11–20-year age group. Regarding residence, a higher proportion of MRSA isolates were from rural areas 9/14 (64.3%) compared to urban areas 5/14 (35.7%) (Table 3).

**TABLE 2:** Antimicrobial susceptibility patterns of *S. aureus* isolate from patients suspected for bacterial infection at Debre Markos Comprehensive Specialized Hospital, Northwest Ethiopia, 2024 (*N* = 38).

Antimicrobials (disk content)^1^	Resistant		Intermediate, *n* (%)	Sensitive	
	*n* (%)	95% CI		*n* (%)	95% CI
Cefoxitin (30 μg)	14 (36.9)	23.4–52.7	–	24 (63.1)	47.3–76.6
Clindamycin (2 μg)	5 (13.1)	5.8–27.3	5 (13.1)	28 (73.8)	58.0–85.0
Erythromycin (15 μg)	9 (23.7)	13.0–39.2	6 (15.8)	23 (60.5)	44.7–74.4
Trimethoprim/sulfamethoxazole (1.25/23.75 μg)	9 (23.7)	13.0–39.2	5 (13.5)	24 (63.2)	47.3–76.6
Azithromycin (15 μg)	2 (5.3)	1.5–17.3	4 (10.5)	32 (84.2)	69.6–92.6
Tetracycline (30 μg)	13 (34.2)	21.2–50.1	4 (10.5)	21 (55.3)	39.7–69.9
Ciprofloxacin (5 μg)	4 (10.5)	4.2–24.1	7 (18.5)	27 (71.0)	55.2–83.0
Gentamicin (10 μg)	16 (42.1)	27.9–57.8	4 (10.5)	18 (47.4)	32.5–62.7
Penicillin (10 U)	19 (50.0)	34.8–65.2	–	19 (50.0)	34.8–65.2
Nitrofurantoin (30 μg)	2 (5.2)	1.5–17.3	11 (29.0)	25 (65.8)	49.9–78.8

^1^CLSI, 2022.

**TABLE 3:** Methicillin susceptibility pattern of *S. aureus* against sociodemographic characteristics among patients suspected for bacterial infection at Debre Markos Comprehensive Specialized Hospital, Northwest Ethiopia, 2024 (*N* = 38).

Socio-demographic variables		Methicillin susceptibility pattern				Total (*N* = 38)
		MRSA (*n* = 14)		MSSA (*n* = 24)		
		*n* (%)	95% CI	*n* (%)	95% CI	
Sex	Male	9 (64.3)	52.2–79.2	14 (54.1)	41.0–77.7	23 (60.5)
	Female	5 (35.7)	25.2–48.3	10 (45.9)	41.8–74.8	15 (39.5)
Age group	< 1 year	1 (7.15)	6.1–21.2	2 (8.3)	4.8–24.6	3 (7.9)
	1–10 years	1 (7.15)	6.1–21.2	2 (8.3)	4.8–24.6	3 (7.9)
	11–20 years	0 (0)	0.1–20.3	1 (4.2)	2.6–16.2	1 (2.6)
	21–30 years	2 (14.3)	10.7–21.0	4 (16.7)	12.9–30.0	6 (15.8)
	31–50 years	6 (42.8)	34.4–57.4	8 (33.3)	22.1–44.6	14 (36.8)
	≥ 51 years	4 (28.6)	21.2–40.6	7 (29.2)	17.9–35.1	11 (29.0)
Residence	Urban	5 (35.7)	26.3–41.2	9 (37.5)	24.8–52.7	14 (36.8)
	Rural	9 (64.3)	51.2–70.3	15 (62.5)	42.6–78.9	24 (63.2)
Types of specimens	Blood	5 (35.7)	19.3–51.0	7 (29.2)	21.4–42.1	12 (31.6)
	Urine	3 (21.4)	12.1–34.6	6 (25.0)	19.4–37.9	9 (23.7)
	Wound	4 (28.6)	18.9–38.3	5 (20.8)	16.7–31.1	9 (23.7)
	Eye swab	2 (14.3)	11.8–30.9	3 (12.5)	8.9–28.2	5 (13.1)
	CSF	0 (0)	0.2–27.3	1 (4.2)	2.6–35.3	1 (2.6)
	Others	0 (0)	0.3–25.8	2 (8.3)	4.2–20.5	2 (5.3)
Total		14 (36.8)	23.4–52.7	24 (63.2)	47.3–76.6	38 (100)

Others: ear swab, synovial fluid, pleural fluid; CSF, cerebrospinal fluid; MRSA, methicillin resistant *S. aureus*; MSSA, methicillin susceptible *S. aureus*.

Of total isolates from blood, 5/12 (41.7%) were MRSA and 7/12 (58.3%) MSSA. A total of 9 isolates were obtained from urine, with 3/9 (33.3%) being MRSA and 6/9 (66.7%) MSSA. Wound samples also yielded 9 isolates, including 4/9 (44.4%) MRSA and 5/9 (55.6%) MSSA. Among the isolates from eye swabs, 2/5 (40%) were MRSA and 3/5 (60%) MSSA (Table 3).

**FIGURE 2:**
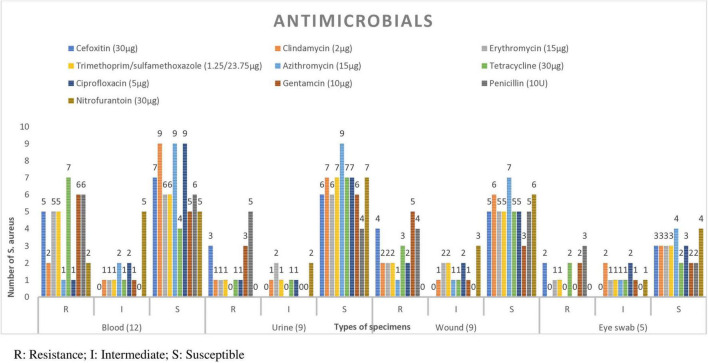
Antimicrobial susceptibility patterns of *S. aureus* isolate in each specimen type from patients suspected for bacterial infection at Debre Markos Comprehensive Specialized Hospital, Northwest Ethiopia, 2024 (*N* = 38). From CSF 1 isolate which was susceptible to all antimicrobials, and 2 isolate form ear, pleural fluid and synovial fluid which were susceptible to al antimicrobials.

Among the isolates from blood specimens, high resistance was observed to tetracycline 7/12 (58.3%) and penicillin 6/12 (50%). In contrast, the highest susceptibility rates were recorded for azithromycin 9/12 (75%), ciprofloxacin 9/12 (75%), and clindamycin 9/12 (75%). Similarly, among isolates from urine samples, notable resistance was seen to penicillin 5/9 (55.6%) and gentamicin 3/9 (33.3%). However, ciprofloxacin and clindamycin remained effective, with 7/9 (77.8%) of isolates to each. Among isolates from wound swabs 4/9 (44.4%) were resistant to cefoxitin, suggesting MRSA (Figure 2).

### 3.4 Inducible clindamycin and multidrug resistance (MDR) patterns of *S. aureus*

The overall observed level of MDR *S. aureus* in this study was 10/38 (26.3%) isolates. Among these isolates, MRSA exhibited a higher level of MDR 8/14 (57.1%) compared to MSSA, 2/24 (8.3%) isolates (Table 4). In total, 14 MRSA and 24 MSSA isolates were examined for clindamycin susceptibility testing. Inducible MLSB resistance (iMLSB) was observed in 3/14 (21.4%) MRSA isolates and 7/24 (29.1%) MSSA isolates, accounting a total of 10/38 (26.3%) isolates. Similarly, constitutive MLSB resistance (cMLSB) was detected in 2/14 (14.3%) MRSA isolates and 3/24 (12.5%) MSSA isolates, making up 5/38 (13.1%) isolates overall. The MS phenotype was found in 4/14 (28.6%) MRSA isolates and 1/24 (4.2%) MSSA isolate, totaling 5/38 (13.1%) isolates. The sensitive (S) phenotype was the most common, identified in 5/14 (35.7%) MRSA isolates and 13/24 (51.2%) MSSA isolates, for a total of 18/38 (47.4%) isolates (Table 5).

**TABLE 4:** Patterns of multidrug resistance (MDR) of *S. aureus* isolates among study participants suspected for bacterial infection at Debre Markos Comprehensive Specialized Hospital, Northwest Ethiopia, 2024 (*N* = 38).

*S. aureus* (*n*)	Multidrug resistance patterns of *S. aureus*, *n* (%)							
	R0 *N* (%)	R1 *N* (%)	R2 *N* (%)	R3 *N* (%)	R4 *N* (%)	R5 *N* (%)	R6 *N* (%)	R ≥ 3 *N* (%)
MRSA (*n* = 14)	0 (0.0)	0 (0.0)	6 (42.9)	3 (21.4)	2 (14.3)	3 (21.4)	0 (0.0)	8 (57.1)
MSSA (*n* = 24)	13 (54.2)	6 (25.0)	3 (12.5)	1 (4.2)	1 (4.2)	0 (0.0)	0 (0.0)	2 (8.3)
Total (*n* = 38)	13 (34.2)	6 (15.8)	9 (23.7)	4 (10.5)	3 (7.9)	3 (7.9)	0 (0.0)	10 (26.3)

R0, R1, R2, R3, R4, R5, R6, R. ≥ 3: sensitive to all, resistance to one, two, three, four, five, six and greater than or equal to three antibiotics tested, respectively; MDR (R ≥ 3): multidrug resistance (if resistance to greater than or equal to three antibiotics). MSSA: methicillin-susceptible *S. aureus*; MRSA: methicillin-resistant *S. aureus*.

**TABLE 5:** Macrolide-lincosamide-streptogramin B (MLSB) resistance phenotype in MRSA and MSSA among participants suspected for bacterial infection at Debre Markos Comprehensive Specialized Hospital, Northwest Ethiopia, 2024 (*N* = 38).

MLSB resistance phenotype	MRSA (*n* = 14)		MSSA (*n* = 24)		Total, *n* (%)
	*n* (%)	95% CI	*n* (%)	95% CI	
ERY-R, CLN-S; D test positive (iMLSB)^1^	3 (21.4)	7.1–49.0	7 (29.1)	13.7–50.2	10 (26.3)
ERY-R, CLN-R (cMLSB)	2 (14.3)	4.0–39.9	3 (12.5)	2.7–32.4	5 (13.15)
ERY-R, CLN-S; D test negative (MS phenotype)	4 (28.6)	11.7–54.6	1 (4.2)	0.1–21.1	5 (13.15)
ERY-S, CLN-S (S phenotype)	5 (35.7)	17.4–59.1	13 (51.2)	34.7–72.5	18 (47.4)
Total	14 (100)	23.4–52.7	24 (100)	47.3–76.6	38(100)

ERY, erythromycin; CLN, clindamycin; S, sensitive; R, resistant; Constitutive MLSB, constitutive MLSB phenotype; iMLSB, inducible MLSB phenotype; MS, MS phenotype; MRSA, methicillin-resistant *S. aureus*; MSSA, methicillin susceptible *S. aureus*.

^1^Clindamycin should be avoided in cases of inducible MLSB resistance (iMLSB), which can lead to treatment failure if not detected with a D-test.

Clindamycin resistance by disk diffusion test detected in 5/38 (13.1%) isolates. These isolates demonstrate constitutive resistance to clindamycin, likely due to constant expression of resistance genes. On the other hand, inducible clindamycin resistance (iMLSB by D-test) detected in 10/38 (26.3%) isolates. These isolates appear susceptible to clindamycin by routine disk diffusion but show resistance when induced by erythromycin, indicating the presence of erm genes. The D-test identified twice as many resistant isolates as the routine clindamycin disk diffusion (26.3% vs. 13.1%). Without performing the D-test, 10 resistant isolates would be falsely considered susceptible, posing a significant risk of treatment failure (Tables 2, 5).

### 3.5 Factors associated with *S. aureus* infections

Various socio-demographic and clinical characteristics of the study participants were assessed for their potential association with the prevalence of *S. aureus* in patients suspected of bacterial infections. In the bivariable logistic regression analysis, factors such as age, residence, educational level, family size, income level, recent surgery, presence of chronic diseases, duration of hospital stays, and the initiation and discontinuation of medication without physician orders all had a *P*-value of less than 0.25. Significant associations were identified for the following factors: rural residence (COR = 2.13, 95% CI: 1.06–4.29; *P* = 0.033), family size of 4–6 members (COR = 3.59, 95% CI: 1.34–8.92; *P* = 0.006), family size greater than 6 members (COR = 8.81, 95% CI: 1.64–24.15; *P* = 0.010), illiteracy (COR = 10.0; 95% CI: 3.48–28.66; *P* = 0.001), and monthly income of less than 3,000 ETB (COR = 8.42, 95% CI: 2.74–25.79; *P* = 0.001). These factors were considered significant and were included as candidates for multivariable logistic regression analysis (Table 6).

**TABLE 6:** Bivariate and multivariate analysis of associated factors for *S. aureus* infections at Debre Markos Comprehensive Specialized Hospital, 2024 (*N* = 339).

Variables		Culture result		COR (95% CI)	*P*-value	AOR (95% CI)	*P*-value
		Yes *n* (%)	No *n* (%)				
Duration of hospital stay	1 day	5 (3.4)	143 (96.6)	1		1	
	2–4 days	14 (10.6)	118 (89.4)	3.39 (1.19–9.69)	0.023*	7.72 (1.73–30.48)	0.007**
	5–6 days	18 (35.3)	33 (64.7)	15.60 (5.40–25.05)	0.001	24.11 (5.07–41.62)	0.001
	> 1 week	1 (12.5)	7 (87.5)	4.08 (0.42–23.83)	0.226	4.27 (0.25–22.92)	0.315
Educational status	Illiterate	12 (27.9)	31 (72.1)	10.0 (3.48–28.66)	0.001*	13.51 (3.56–21.90)	0.018**
	Primary school	12 (16.0)	63 (84.0)	4.92 (1.77–13.68)	0.002	4.19 (0.65–27.10)	0.132
	Secondary school	8 (13.3)	52 (86.7)	3.97 (1.32–11.98)	0.140	4.05 (0.93–17.58)	0.062
	College and above	6 (3.7)	155 (96.3)	1		1	
Income	< 3,000	20 (25.0)	60 (75.0)	8.42 (2.74–25.79)	0.001*	6.20 (1.03–30.09)	0.046**
	3,001–6,000	14 (91.0)	140 (9.0)	2.52 (0.81–7.89)	0.111	1.35 (0.32–5.71)	0.686
	≥ 6,001	4 (3.8)	101 (96.2)	1		1	
Family size	1–3	6 (4.6)	125 (95.4)	1		1	
	4–6	29 (14.7)	168 (85.3)	3.59 (1.34–8.92)	0.006*	4.3 (1.20–15.39)	0.025**
	> 6	3 (27.3)	8 (72.7)	7.81 (1.64–24.15)	0.010	12.14 (2.38–20.43)	0.024
Alcohol intake	Yes	9 (29.0)	22 (71.0)	3.93 (1.65–9.34)	0.002	2.09 (0.53–8.17)	0.290
	No	29 (9.4)	279 (90.6)	1		1	
Age group	< 1 year	3 (3.9)	73 (96.1)	1		1	
	1–10 year	3 (11.5)	23 (88.5)	3.17 (0.59–16.82)	0.175	6.17 (0.65–18.73)	0.114
	11–20 year	1 (4.7)	23 (95.3)	1.05 (0.10–10.67)	0.962	1.42 (0.10–19.14)	0.789
	21–30 year	6 (11.8)	45 (88.2)	3.24 (0.77–13.62)	0.108	4.53 (0.65–11.22)	0.125
	31–50 year	14 (12.3)	100 (87.7)	3.4 (0.94–12.29)	0.061	2.56 (0.51–12.96)	0.255
	≥ 51 year	11 (22.9)	37 (77.1)	7.23 (1.9–27.53)	0.004*	3.14 (0.55–17.9)	0.196
Having healthcare worker family	Yes	3 (3.2)	91 (96.8)	1		1	
	No	35 (14.3)	210 (85.7)	0.19 (0.06–0.66)	0.008*	2.82 (0.58–13.51)	0.195
Residence	Urban	14 (7.3)	167 (92.7)	1		1	
	Rural	24 (15.2)	134 (84.8)	2.13 (1.06–4.29)	0.033*	3.52 (2.03–12.75)	0.021**
Catheter use	Yes	3 (30.0)	7 (70.0)	3.6 (0.89–14.55)	0.072*	0.96 (0.13–7.17)	0.972
	No	35 (10.6)	294 (89.4)	1		1	
Recent surgery	Yes	8 (19.0)	34 (81.0)	2.09 (0.88–4.93)	0.091*	0.91 (0.23–3.54)	0.896
	No	30 (10.1)	267 (89.9)	1		1	
Illness in the past 4 weeks	Yes	37 (12.4)	262 (87.6)	5.51 (0.73–31.29)	0.097*	0.69 (0.06–8.00)	0.772
	No	1 (2.5)	39 (97.5)	1		1	
Presence of chronic diseases	Yes	6 (20.0)	24 (80.0)	2.16 (0.82–5.69)	0.117*	1.21 (0.28–5.12)	0.797
	No	32 (10.3)	277 (89.7)	1		1	

*There was association, so the variables were candidates for multivariate logistic regression; 1$ = 131.6 ETB, 1 = Reference variable; **There was significant association.

In the multivariable logistic regression analysis of factors associated with *S. aureus* prevalence, residence, educational level, family size, income level, and duration of hospital stay showed significant associations (*P* < 0.05). The odds for infection with *S. aureus* were 12 times more among participants with a family size greater than six compared to those with smaller families (AOR = 12.14, 95% CI: 2.38–20.43; *P* = 0.024). The odds for infection with *S. aureus* were 6.2 times higher among patients with a monthly income of less than 3,000 ETB compared to those earning more than 3,000 ETB (AOR = 6.20; 95% CI: 1.03–30.09; *P* = 0.046). Similarly, the odds for infection with *S. aureus* were significantly higher among individuals residing in rural areas (AOR = 3.53; 95% CI: 2.03–12.75; *P* = 0.021). In addition, participants who were illiterate had 13.51 times higher odds for *S. aureus* infection than those who could read and write (AOR = 13.51; 95% CI: 3.56–21.90; *P* = 0.018). However, factors such as age group, recent surgery history, presence of chronic diseases, duration of hospital stay, and changes in medication without a physician’s order were not significantly associated with *S. aureus* infection (*P* > 0.05) (Table 6).

## 4 Discussion

*Staphylococcus aureus* is a significant human pathogen capable of causing both nosocomial and community-acquired infections (Guo et al., 2020). Based on the current findings, the overall prevalence of *S. aureus* was 11.2% (95% CI: 8–15). This result is lower than those reported in Arba Minch, Ethiopia (49.7%) (Mama et al., 2019), University of Gondar (17%) (Ambachew et al., 2022), and a study conducted in Nepal in 2018 (16.1%) (Adhikari et al., 2017). The discrepancies in prevalence rates may be attributed to geographical variations, socioeconomic factors, and differences in the types of samples collected. However, the current findings are consistent with those from the Amhara region of Ethiopia (10.2%) (Moges et al., 2023) and another study in Nepal (11.8%) (Thapa et al., 2021).

The prevalence of *S. aureus* isolates from different clinical samples was analyzed, revealing that blood samples accounted for the highest proportion of *S. aureus* isolation at 12/38 (31.6%). This finding is higher than studies conducted in Gondar (19.9%) (Ambachew et al., 2022), Egypt (21.5%), and Nepal (22.2%) (Adhikari et al., 2017). The observed differences may be attributed to variations in healthcare system. However, this prevalence is lower than the results from a study conducted in the Amhara region of Ethiopia in 2022, which reported a prevalence of 43.9% (Moges et al., 2023). This discrepancy may be due to the diverse nature of the samples, which were collected from various anatomical sites. In this study, the most common clinical specimen for *S. aureus* isolates was blood 31.6%, followed by wound samples 23.7%. This pattern is consistent with findings from the Amhara region of Ethiopia, where blood samples accounted 43.9% of isolates, followed by wound samples 32.4% (Moges et al., 2023).

The antimicrobial susceptibility testing of 38 bacterial isolates, azithromycin exhibited the highest sensitivity at 84.2%, followed by clindamycin (73.8%) and ciprofloxacin (71.0%). Conversely, penicillin and gentamicin showed the highest resistance rates, at 50.0% and 42.1%, respectively. When comparing these findings to other studies in Ethiopia, variations in antimicrobial resistance patterns are evident. For instance, a five-year retrospective analysis at the Ethiopian Public Health Institute reported that *S. aureus* exhibited very high resistance to penicillin G (86.7%) and ciprofloxacin (50%) (Abdeta et al., 2023).

The frequency of methicillin-resistant *S. aureus* in the current study was found to be 36.9%. This prevalence is comparable to findings from various locations in Ethiopia and beyond, including: DMCSH, Ethiopia (28.3%) (Tefera et al., 2021), Gondar, Ethiopia (21.2%), pooled study in the Amhara region, Ethiopia (34.5%) (Moges et al., 2023), Dessie, Ethiopia (30.4%) (Ambachew et al., 2022), Libya (21.4%) (Wareg et al., 2014) and Pakistan (36.1%) (Ullah et al., 2016). The relatively high prevalence of MRSA in this study may be attributed to low socioeconomic status, rural residence and illiteracy. In contrast, the current finding is lower than the global prevalence and trend of *S. aureus* infections reported in 2016, which was 40.3% (Diekema et al., 2019), and the prevalence observed in Arba Minch, Ethiopia, which was significantly higher at 82.3% (Mama et al., 2019). However, it is noteworthy that this finding is higher than that reported in a study conducted at Yekatit 12 Hospital, Ethiopia, where the prevalence of MRSA was 17.5% (Dilnessa and Bitew, 2016).

In this study, inducible clindamycin (iMLSB) resistance was detected in 26.3% of *S. aureus* isolates, with 21.4% of MRSA and 29.1% of MSSA isolates exhibiting the phenotype. These findings are comparable to reports by Fiebelkorn et al. (2003) who observed iMLSB resistance in 19% of MRSA and 28% of MSSA isolates, in Gondar, Ethiopia (25.8%) (Ambachew et al., 2022), Arba Minch, Ethiopia (24%) (Mama et al., 2019). However, it is slightly higher than reports in systematic review in Africa 19.8% (Assefa, 2022), in Egypt 13.6% (Kishk et al., 2020), and Yekatit 12 Hospital, Ethiopia 11.9% (Dilnessa and Bitew, 2016). Further, higher rates have been reported in other regions, such as by Ciraj et al. (2009) who found iMLSB resistance in 40% of MRSA isolates. These variations may reflect regional antibiotic usage patterns and local epidemiology.

Clindamycin resistance detected by the routine disk diffusion method was observed in 5/38 (13.1%) isolates, indicating constitutive resistance. This form of resistance is typically due to the continuous expression of *erm* genes encoding methyltransferases that alter the 23S rRNA target site, rendering macrolide-lincosamide-streptogramin B (MLSB) antibiotics ineffective (Leclercq, 2002). In contrast, inducible clindamycin resistance (iMLSB) was detected in 10/38 (26.3%) isolates using the D-test, which is designed to uncover resistance that is not apparent in standard susceptibility testing. These isolates appear clindamycin-susceptible by disk diffusion, but express resistance when erythromycin acts as an inducer, reflecting the presence of inducible *erm* gene expression (Fiebelkorn et al., 2003). This notable difference underscores the importance of performing the D-test alongside routine antimicrobial susceptibility testing. Without it, inducible resistant strains may be misclassified as susceptible, potentially leading to clinical treatment failure (CLSI, 2022). Our findings highlight that inducible resistance may be twice as prevalent as constitutive resistance (26.3% vs. 13.1%), a trend consistent with reports from similar settings (Ciraj et al., 2009).

The prevalence of MDR was 26.3% in the current study. This rate is significantly lower than those reported in several other studies, including Amhara regional state reports 72.7% (Moges et al., 2023), Yekatit 12 Hospital 50.5% (Dilnessa and Bitew, 2016), Eritrea 39.5% (Garoy et al., 2019), and Saudi Arabia 47% (Albarrag et al., 2020). Conversely, the MDR rate in this study was higher than the previous study conducted at DMCSH, which reported a rate of 21.7% in 2020 (Tsige et al., 2020). The observed differences in MDR rates across studies may be attributed to differences in antimicrobial stewardship practices, sample sizes, infection control measures, patient populations, and geographic locations. Factors such as the availability and misuse of antibiotics, differences in diagnostic capabilities, and time of study conduct may also contribute. Additionally, variations in study design and the criteria used to define MDR could influence reported rates.

Regarding the prevalence of *S. aureus* and its association with socio-demographic and clinical data, the majority of the isolates were recovered from male patients, accounting 57.9% isolates. The age distribution showed that 36.8% patients were aged between 31 and 50 years, followed by 29.0% patients who were older than or equal to 51 years. Additionally, a significant number of isolates were obtained from rural residents 64.1%, with a notable prevalence among farmers 52.6%. These findings are consistent with a study conducted in India, which reported that 62/97 (63.9%) *S. aureus* isolates were from male patients, while 35/97 (36.1%) isolates were from female participants. The highest number of isolates in that study was also found in the age group of 31–50 years, comprising 32% of the total isolates (Lall and Sahni, 2014).

According to a study conducted in Gondar, patients with a family size of 4–6 and those with a family size greater than six were found to be more likely to have *S. aureus* infections compared to patients with a family size of 1–3 (Ambachew et al., 2022). This finding aligns with our results, which indicate that study participants with a family size greater than six were 12.14 times more likely to have *S. aureus* infections (*P* = 0.024) than those with a family size of one up to three. The variations between our findings and those of other studies can be attributed to several factors, including differences in sample sizes, seasonal variations affecting infection rates, variations in infection control practices, demographic characteristics of the study populations, and history of prior antibiotic usage, which may influence resistance patterns and infection rates (Magill et al., 2014). These factors can play a significant role in producing discordant results in studies conducted in different regions of the world.

Several factors may explain the association between inability to read and write, rural living, and increased risk of infection. Individuals who are illiterate may have limited access to health information regarding hygiene practices, wound care, and infection prevention, making them less likely to recognize early signs of infection or to properly follow medical advice. In rural areas, access to clean water, soap, and basic sanitation is often limited, increasing the risk of skin infections such as those caused by *S. aureus*. Additionally, rural communities typically have fewer healthcare facilities and providers, resulting in delayed or inadequate treatment of minor wounds or infections, which can allow *S. aureus* to establish infections (Peters et al., 2008). Moreover, without proper medical guidance, antibiotics may be misused- either overused, underused, or used improperly- contributing to the persistence and severity of infections (Laxminarayan et al., 2013). Overcrowded living conditions, often seen in rural households, can also facilitate the spread of *S. aureus* (David and Daum, 2010). Finally, malnutrition, which is more prevalent in impoverished rural areas, may weaken the immune system, making individuals more susceptible to infections (Katona and Katona-Apte, 2008).

## 5 Conclusion

The high occurrence of MRSA and iMLSB phenotypes among *S. aureus* highlights the importance of including methicillin resistance testing and the D-test in routine susceptibility screening to ensure effective management. The D-test identified twice as many resistant isolates as the routine clindamycin disk diffusion test. Moreover, key socio-demographic factors such as illiteracy, larger family size, and lower income levels were significantly associated with *S. aureus* infection, highlighting potential socioeconomic determinants of health vulnerability. Targeted health education, particularly for low-literacy populations, is essential to raise awareness about hygiene and antibiotic misuse. Strengthening routine screening for *S. aureus* and resistance patterns, along with implementing robust antibiotic stewardship programs, is critical for early detection and control. Additionally, addressing underlying socioeconomic factors-such as improving income and living conditions-can help reduce vulnerability to infection. Future research should investigate the genetic mechanisms underlying resistance and evaluate interventions to reduce MRSA and iMLSB prevalence.

### 5.1 Limitations of the study

This study was performed on phenotypic antimicrobial susceptibility testing among isolates. The genotypic tests for inducible clindamycin resistance such as erm and mec A gene detection were not performed. The dilution effect of use of different types of specimens would lower the prevalence of *S. aureus*. Additionally, the use of convenience sampling and hospital-based design may limit generalizability.

## Data Availability

The raw data supporting the conclusions of this article will be made available by the authors, without undue reservation.
